# Using large-scale respondent driven sampling to monitor the end of an HIV epidemic among persons who inject drugs in Hai Phong, Viet Nam

**DOI:** 10.1371/journal.pone.0259983

**Published:** 2021-11-18

**Authors:** Don C. Des Jarlais, Kamyar Arasteh, Duong Thi Huong, Khuat Thi Hai Oanh, Jonathan P. Feelemyer, Pham Minh Khue, Hoang Thi Giang, Nham Thi Tuyet Thanh, Vu Hai Vinh, Sao Mai Le, Roselyne Vallo, Catherine Quillet, Delphine Rapoud, Laurent Michel, Didier Laureillard, Jean Pierre Moles, Nicolas Nagot, For The DRIVE Study Team

**Affiliations:** 1 New York University School of Global Public Health, New York, NY, United States of America; 2 Haiphong University of Medicine and Pharmacy, Haiphong, Vietnam; 3 Supporting Community Development Initiatives, Hanoi, Vietnam; 4 Dept of Infectious and Tropical diseases, Viet Tiep Hospital, Haiphong, Vietnam; 5 Pathogenèses and control of chronic and emerging infections, University of Montpellier, Inserm, Etablissement Français du Sang, University of Antilles, Montpellier, France; 6 Pierre Nicole Center, French Red Cross, CESP/Inserrm 1018, Paris, France; 7 Infectious Diseases Department, Caremeau University Hospital, Nîmes, France; Centers for Disease Control and Prevention, UNITED STATES

## Abstract

**Aims:**

To describe the use of large-scale respondent driven sampling (RDS) surveys to demonstrate the “end of an HIV epidemic” (HIV incidence < 0.5/100 person-years) among persons who inject drugs (PWID) in a middle-income country. Large sample sizes are needed to convincingly demonstrate very low incidence rates.

**Methods:**

4 large surveys (Ns approximately 1500 each) were conducted among PWID in Hai Phong, Vietnam in 2016–2019. Respondent driven sampling (RDS) with a modification to add snowball sampling was used for recruiting participants. HIV incidence was measured through recency testing, repeat participants across multiple surveys and in a cohort study of PWID recruited from the surveys. RDS analytics (time to equilibria and homophilies for major variables) were used to assess similarities/differences in RDS only versus RDS plus snowball recruiting. Characteristics were compared among respondents recruited through standard RDS recruitment versus through snowball sampling. An overall assessment of the robustness of RDS to modification was made when adding a snowball sampling recruitment.

**Results:**

RDS recruiting was very efficient in the first 5 weeks of each survey with approximately 180 respondents recruited per week. Recruiting then slowed considerably, and snowball sampling (permitting an individual respondent to recruit large numbers of new respondents) was added to the existing RDS recruiting. This led to recruiting within 13–14 weeks of 1383, 1451, 1444 and 1268 respondents, close to the target of 1500 respondents/survey.

Comparisons of participants recruited through standard RDS method and respondents recruited through snowball methods showed very few significant differences. RDS analytics (quickly reaching equilibria, low homophilies) were favorable for both RDS recruited and total numbers of participants in each survey. DRug use and Infections in ViEtnam (DRIVE) methods have now been officially adopted in other provinces.

**Conclusions:**

RDS appears to be quite robust with respect to adding a modest number of participants recruited through snowball sampling. Large sample sizes can provide compelling evidence for “ending an HIV epidemic” to policy makers in a PWID population in a middle income country setting.

## Introduction

People who inject drugs (PWID) have traditionally been considered a “hard to reach” or “hidden” population. The illicit nature of their drug use, the stigmatization of injecting drug use, and the stigmatization of some of the adverse consequences of injecting drug use create many reasons for PWID to conceal their drug use from law enforcement, society in general, health service providers, and friends and family.

HIV infection is one of the most important adverse consequences of injecting drug use, and has been a leading cause of death among PWID in many countries. We now have the public health tools, “combined prevention and care,” including syringe access programs, medication-assisted treatment (MAT) for opioid use disorders, and anti-retroviral treatment (ART) for HIV infection for controlling HIV among PWID [1]. When implemented at high coverage levels, combined prevention and care has been quite effective in achieving very low HIV incidence rates (< 1/100 person-years), thereby effectively “ending HIV epidemics” among PWID in many high-income settings [2]. Additionally, the DRug use and Infections in ViEtnam (DRIVE) study recently observed a very low HIV incidence rate of 0.085/100 person years at risk (PYAR)(95% CI 0.02–0.25/100 PYAR) among PWID in Hai Phong, Vietnam, a middle-income setting [3].

Four large scale modified respondent driven sampling (RDS) surveys were central in obtaining the data needed to assess the end of the HIV epidemic among PWID in the DRIVE study. For many reasons, RDS has been a very frequently used method for recruiting PWID for research studies [4–6]. It can be both efficient and cost-effective for recruiting large numbers of research participants, and may provide an approximation of a representative sample of PWID [7], though this is debated in the literature [8,9]. In addition to the debate over the extent to which RDS generates representative samples of PWID populations, there is continuing study of implementation issues, such as the best characteristics of seeds, the number of seeds, the density of social networks, managing recruitment flow, as well as cost effectiveness issues [8,10].

We believe that the four modified RDS surveys in the DRIVE study (Ns = 1383, 1451, 1444 and 1268) represent the most extensive use of RDS with PWID in a single city. We report here on: (1) the implementation of the RDS surveys; (2) the need for modifying the standard RDS methods in order to achieve large sample sizes; (3) the use of RDS analytics (reaching equilibria, homophilies) for comparing RDS to a hybrid recruitment using RDS plus snowball sampling; (4) consider whether the surveys were likely to be “representative” or “biased”; and (5) consider the “robustness” of RDS methods to modifications of the standard procedures.

## Background: HIV among PWID in Hai Phong, Vietnam

Heroin injection was introduced into Vietnam in the latter part of the twentieth century [11,12] and was followed by an HIV epidemic among PWID in the early part of the twenty-first century [13]. Hai Phong had experienced a high HIV epidemic among PWID, with seroprevalence reaching 66% in 2006 [14]. With the strong support of the local provincial government and international donors, Hai Phong has also been in the forefront of implementing evidence-based combined prevention programs for PWID in Viet Nam. Syringe distribution/exchange was initiated in 2007, methadone maintenance pilot programs in Ho Chi Minh City and Hai Phong began in 2008, and ART for all HIV infected persons (regardless of CD4 cell count) was initiated in 2014.

The DRIVE study was undertaken to determine if “combined prevention and care” for HIV could “end the HIV epidemic” among PWID in the city of Hai Phong, Viet Nam. One component of “ending the HIV epidemic” was reducing HIV incidence to less than 0.5/100 PYAR [15].

## Materials and methods

### Research team

DRIVE has been conducted by a multi-national team of researchers from Hai Phong University of Medicine and Pharmacy in Viet Nam, the University of Montpellier, France, and New York University (NYU), USA. (The US team was previously affiliated with the Icahn School of Medicine at Mount Sinai and later with the NYU School of Global Public Health in New York City). Most importantly, the team included members of community-based organization (CBO) -peer support groups in Hai Phong affiliated with Supporting Community Development Initiatives (SCDI). The CBOs were fully integrated into the research team—they participated in research decisions and data collection (by conducting interviews with PWID) and provided support and assistance to the participants in the study.

### Preliminary feasibility study

A DRIVE-IN RDS survey of 603 respondents was conducted in 2014 to assess the feasibility of the planned research methods and of the reaching the goal of ending the epidemic among PWID in Hai Phong [4,16]. The DRIVE-IN RDS survey provided the needed knowledge of the PWID population for planning the larger RDS surveys used in DRIVE. Based on the 2014 data, the estimated HIV incidence was 1-2/100 PYAR [16].

### RDS surveys

We conducted four large-scale RDS surveys among PWID, in 2016 (RDS1), 2017 (RDS2), 2018 (RDS3) and 2019 (RDS4). The target number of participants for each survey was 1500. The eligibility criteria for participation in the surveys were: age 18 or greater, recent injection drug use (validated through urinalysis of an injectable drug and inspection for recent injection marks), residence in Hai Phong, and ability to provide informed consent.

We began each of these surveys using standard RDS methods, [17,18]. Each survey began with the selection of 20 “seeds,” 10 at each of the two research sites. The seeds were selected by CBO staff for diversity (age, gender, sexual orientation, HIV status, geographic location in Hai Phong) and having large social networks among PWID. Each seed first participated in all study procedures, and then was given 3 numbered coupons for recruiting new survey participants. The numbered coupons contained the study logo, the addresses of the two research sites, the hours of site operation, and the dates for which the coupons were valid. New participants needed to bring study coupons to the study sites to participate. After they participated, these new participants were then given numbered coupons for recruiting additional participants. Participants were paid modest honoraria for both their own time and effort in the study (150,000 Vietnamese dong (VND), approximately US $7.50) and for recruiting new participants (50,000 VND per recruit, approximately US $2.50). The numbered coupon manager system permitted tracking the participants eligible for the recruitment honoraria.

After informed consent, participants were first screened for eligibility, including urinalysis positive for an injectable drug (heroin or methamphetamine) and skin marks for recent injecting. A fingerprint reader was used to prevent multiple participation in a single survey and to track participants across multiple surveys. A structured interview was administered by a trained interviewer to collect information related to demographic characteristics, drug use and sexual behavior, and use of HIV-related services. Blood samples were taken for HIV and HCV testing. Among those that were HIV positive, we collected CD4 cell counts, ART medication adherence, and HIV viral load. Blood samples were also stored for future possible analyses.

All HIV seropositive RDS participants and a sample of the HIV seronegative participants were invited to participate in cohort studies with follow-up interviews every 6 months to assess changes in the use of health-related services and for changes in HIV and HCV status. The participants in the cohort studies also received assistance from the CBOs in accessing health-related services and support for adherence to methadone treatment and ART.

Approximately five weeks into each of the RDS surveys, recruitment of new participants slowed considerably. The CBO staff reported that participants were having considerable difficulty recruiting new participants. Since the pilot study in 2014, police surveillance of drug distribution sites (“hotspots”) had increased, making it much more difficult for study participants to recruit new participants. There were also some PWID who lived very far from the study sites, so that it would not be logistically feasible for them to travel to the study site to participate in DRIVE, and others were unable to participate due to work commitments.

These challenges led to lower than expected recruitment using only RDS methods after the first five weeks in each survey. We were therefore forced to deviate from standard RDS methods. Rather than give a set number of recruitment coupons to every participant, we interviewed participants about their ability to recruit new participants—if they stated that they would not be able to recruit new participants, we did not give them recruiting coupons; if they said that they would be able to recruit large numbers of new participants, we gave them up to 20 coupons each. This was an addition of “snowball” recruiting in which participants are permitted to recruit large numbers of new participants [19,20], to the standard RDS methods.

We identified subjects recruited through standard RDS methods (who would have been recruited with the standard limit of 3 recruiting coupons) versus respondents recruited through snowball sampling (where an individual participant could recruit up to 20 additional participants) for comparisons of demographic characteristics, drug use behaviors and HIV and HCV status. The first three subjects recruited by an individual participant were classified as RDS recruited and new participants beyond the initial 3 who were recruited by an individual participant were classified as snowball recruits. (A critical assumption within RDS is that a recruiter will randomly recruit new respondents from within their injecting network [21,22]. Thus, our classification was simply an easy method for consistently classifying the RDS versus the snowball sampling respondents when a respondent recruited more than 3 new respondents.)

We calculated reaching equilibrium (the point in the succeeding waves of data collection where the value for a variable does not change with further data collection and is an indication that the survey is no longer dependent upon the characteristics of the seeds) [23] and “homophilies” (the likelihoods that participants recruit persons similar to themselves) for gender, HIV, HCV, and median age in each of the 4 RDS surveys. We also calculated “homophilies” for use of methadone (as measured by urinalysis) and use of methamphetamine (measured by self-report) [23]. We selected these variables for their relevance to HIV infection and on the basis that they would be minimally influenced by social desirability bias. (The use of methamphetamine in the preceding 6 months was higher in the self-report data than in the intake urinalysis.) We did not include injecting risk behavior (syringe sharing) as a major variable because of the high probability of social desirability bias and because this behavior was uniformly low (reported by less than 5% of PWID) in all of the surveys.

These RDS analytics were calculated separately for the first 5 weeks of recruitment, when standard RDS methods were used exclusively, and for the entire samples, in which mixed RDS and snowball recruiting methods were used.

As an exploratory analysis, we also calculated RDS weights for respondents recruited within the first 5 weeks of each survey and for the entire samples of subjects recruited in each survey, with mixed RDS and snowball sampling methods.

STATA 15 [24] was used for data analysis. The study was reviewed and approved by the Institutional Review Boards of the Hai Phong University of Medicine and Pharmacy, the Icahn School of Medicine at Mount Sinai and the New York University School of Medicine.

## Results

### Implementation of RDS1, RDS2, RDS3, and RDS4

**Fig 1:**
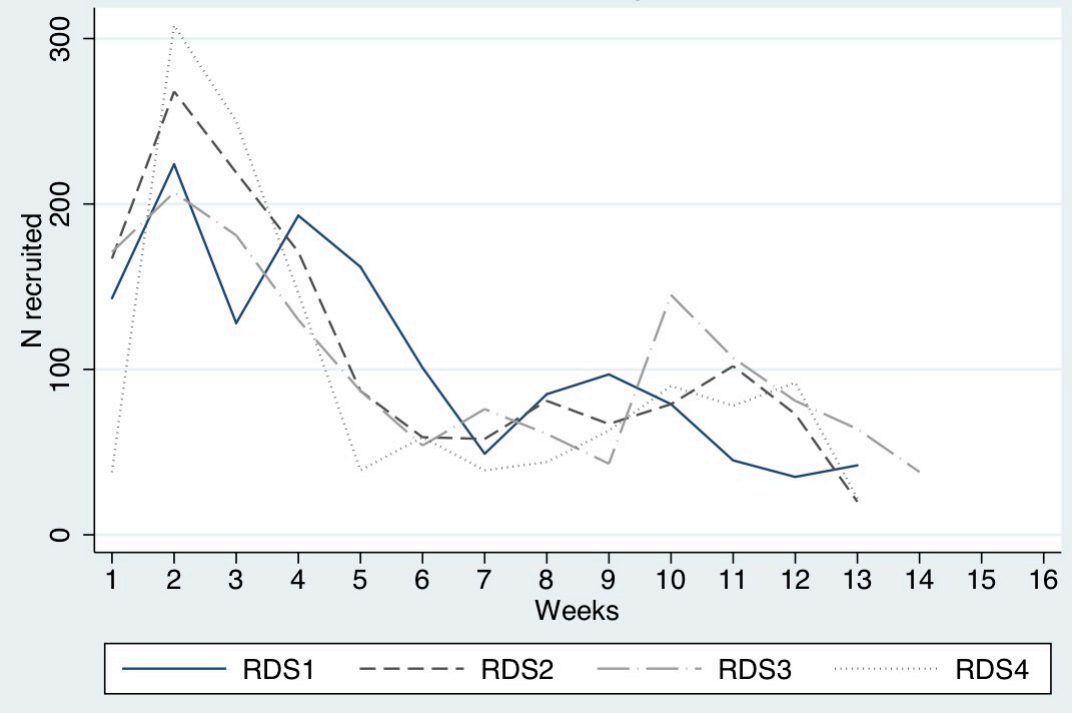
Weekly RDS recruitment of PWID in Hai Phong, Vietnam, 2016–2019*.

1383, 1451, 1444 and 1268 participants were recruited in the RDS1 (in 2016), RDS2 (in 2017) RDS3 (in 2018) and RDS4 (in 2019). Fig 1 shows the numbers of subjects recruited in each week of the RDS surveys. There were clear similarities across the four surveys. Recruitment was quite efficient in the first 5 weeks of each survey, with an average of 181, 214, 177 and 243 participants recruited each week within these periods. After approximately 5 weeks, however, recruitment slowed considerably in each survey, and fell to an average of 96, 76, 88 and 68 participants per week respectively for the next several weeks. As noted in the Methods, we then added snowball sampling which led to increases in the numbers of participants per week, but by week 11 the numbers of participants were again declining, and recruitment was discontinued between weeks 13 and 14. By that time, we had approached the target of 1500 in each survey.

Table 1 presents demographic characteristics, drug use behaviors, HIV and HCV status, for the respondents recruited in the 4 surveys. (As noted above, all respondents reported injecting heroin, verified by urinalysis and inspection of injection marks, as part of the eligibility requirements.) Gender, HCV prevalence, and methamphetamine use remained relatively stable across the 4 surveys. The median age increased from 39 in 2016 to 42 in 2019 (p<0.001). HIV seroprevalence declined modestly but significantly (from 30% in 2016 to 26% in 2019, p = 0.02). Methadone use, measured through urinalysis, increased substantially across the 4 surveys (from 42% in 2016 to 61% in 2019, p<0.001).

**Table 1:** Demographic characteristics, drug use and sexual behaviors and HIV and HCV serostatus among PWID in Haiphong, Viet Nam, 2016–2019*.

Characteristics	RDS1		RDS2		RDS3		RDS4	
Average Age (SD)*	39(9)		40(9)		42(9)		42(8)	
	**N**	**%**	**N**	**%**	**N**	**%**	**N**	**%**
**Total**	1383	100	1451	100	1444	100	1268	100
**Gender**								
Females	83	6	66	5	77	5	72	6
Males	1294	94	1381	95	1362	94	1193	94
Methadone (urine)*								
No	800	58	629	43	521	36	490	39
Yes	582	42	822	57	923	64	778	61
**Methamphetamine (smoked)**								
No	738	53	843	58	812	56	758	60
Yes	645	47	608	42	632	44	510	40
**Unsafe sex with primary partner**								
No	894	65	947	65	878	61	848	67
Yes	489	35	504	35	566	39	420	33
**Unsafe sex with casual partner**								
No	1343	97	1380	95	1401	97	1228	97
Yes	40	3	71	5	43	3	40	3
HIV serostatus*								
Negative	968	70	1061	74	1048	73	939	74
Positive	411	30	375	26	382	27	329	26
**HCV serostatus**								
Negative	407	30	446	32	404	29	344	27
Positive	972	70	964	68	984	71	923	73

* Significant trend (p <.05). Cell counts and percentages may not always add to totals due to missing data.

### Demographics and drug use behaviors

Table 2 presents information on subjects recruited through standard RDS methods and subjects recruited through snowball sampling in each of the 4 surveys. Again, the first 3 new participants recruited by an individual to present at a research site were classified as RDS recruits and any new participants beyond the first 3 were classified as snowball recruits. There were only 2 statistically significant (p < 0.05) differences in the 32 comparisons, a result that is very close to what would be expected by chance.

**Table 2:** Demographics and drug use characteristics for each RDS by whether the participant was recruited by a person with more than 3 recruits (RDS vs. Snowball)*.

	RDS1				RDS2				RDS3				RDS4			
	RDS		Snowball		RDS		Snowball		RDS		Snowball		RDS		Snowball	
Average age (SD)	39(9)		40(9)		40(9)		38(8)		41(9)		43(9)*		42(8)		42(9)	
	N	%	N	%	N	%	N	%	N	%	N	%	N	%	N	%
Total	1269	100	114	100	1319	100	132	100	1245	100	199	100	1155	100	113	100
Gender																
Females	79	6	4	4	61	5	5	4	68	5	8	4	65	6	7	6
Males	1184	94	110	96	1254	95	127	96	1172	95	191	96	1087	94	106	94
Methadone (urine)																
	535	42	47	41	742	56	80	61	795	64	128	64	713	62	65	58
Methamphetamine (smoked)																
	589	46	56	49	548	42	60	45	548	44	84	42	453	39	57	50*
Sharing syringes																
	61	5	7	6	48	4	2	2	42	3	7	4	22	2	1	1
Unsafe sex with primary partner																
	448	35	41	36	461	35	43	33	495	40	71	36	383	33	37	33
Unsafe sex with casual partner																
	34	3	6	5	65	5	6	5	34	3	9	5	38	3	2	2
HIV Seropositive																
	369	29	42	37	343	26	32	24	328	27	54	28	287	25	30	27
HCV Seropositive																
	894	71	78	68	879	68	85	67	852	70	132	70	802	72	68	61*
ART among HIV positives																
	195	91	21	100	254	96	24	100	276	93	51	96	254	97	28	97

* Cell counts and percentages may not always add to totals due to missing data. Significant difference between snowball recruited and RDS recruited (p < 0.05; by t-test for continuous variables, or by chi-square test for proportions).

### Reaching equilibria on major variables

Table 3 presents the number of waves to reach equilibrium for our major variables during the first 5 weeks of recruiting and during the entire recruiting periods. The number of waves were identical for almost all variables in almost all of the surveys with no more than a one wave difference for any variable. Large differences in the number of waves, with the full samples taking more waves to reach equilibrium would indicate that respondents recruited after the first 5 weeks were substantially different from respondents recruited during the first 5 weeks of recruiting.

**Table 3:** Number of waves to equilibrium (whole numbers) followed by homophilies for major variables for first 5 weeks (5w) and for all subjects (full) for major variables in each survey. Homophilies can range between 1.0 to -1.0.

	RDS1 (Full)	RDS1 (5w)	RDS2 (Full)	RDS2 (5w)	RDS3 (Full)	RDS3 (5w)	RDS4 (Full)	RDS4 (5w)
Age	3	3	3	3	3	3	3	3
≤ Median	0.21	0.20	0.25	0.26	0.19	0.16	0.12	0.11
> Median	0.11	0.10	0.15	0.11	0.05	0.02	0.08	0.06
Gender	3	3	4	4	3	3	3	3
Female	0.14	0.16	0.19	0.26	0.08	0.11	0.13	0.06
Male	0.04	-0.1	0.30	0.37	0.28	0.23	0.16	0.14
Methadone	3	3	3	3	4	3	3	3
No	-0.004	-0.03	0.05	0.09	0.13	0.13	0.05	0.11
Yes	0.15	0.13	0.15	0.17	0.22	0.20	0.18	0.12
Methamphetamine	3	3	3	3	3	3	3	3
No	0.08	0.12	0.12	0.11	0.15	0.12	0.06	0.16
Yes	0.22	0.23	0.06	0.04	0.17	0.16	0.21	0.17
HIV	3	3	3	4	3	3	2	3
Negative	0.12	0.17	0.14	0.19	0.08	0.11	0.13	0.10
Positive	0.15	0.13	0.20	0.22	0.18	0.18	0.13	0.09
HCV	2	3	3	3	3	3	3	3
Negative	-0.04	-0.01	0.04	0.05	0.03	0.02	0.10	0.06
Positive	0.01	-0.07	0.09	0.08	0.09	0.08	0.08	0.02

Table 3 also presents the homophilies for the major variables for the entire samples and for the first 5 weeks of recruiting. In all cases, the homophilies are very similar with all comparisons within a difference of 0.10. The homophilies were also generally very close to 0, with only 7 with values greater or less than 0.25. Four of these 7 indicated preferential recruiting by gender, with males more likely to recruit other males in 3 instances and females more likely to recruit females in 1 instance. With the possible exception of gender, there was very little indication of respondents preferentially recruiting new respondents similar to themselves.

#### RDS weighting

As noted in previously published papers, because we did not utilize standard RDS recruitment methods throughout the 4 surveys and because we suspected respondents might be recruiting new respondents outside of their personal networks. Rather, we treated each survey as a convenience sample. In particular, we were concerned that some recruiting may have occurred near methadone programs or near HIV clinics. For this methodological paper, however, we did examine RDS weighting for the surveys. Table 4 presents the RDS weights for each of major variables during the full recruiting and for the first 5 weeks recruiting for the 4 surveys. The weights were almost identical for the full surveys and for the first 5 weeks of recruiting for each of the variables in each of the surveys, and almost all weights were close to 1.0. Weights near 1.0 indicate that the variable is not associated with the reported size of the social network of which the respondent is a member. Using RDS weighting from either the first 5 weeks of recruiting or from the total samples would not have appreciably differed from using the unweighted values in the statistical analyses of these surveys.

**Table 4:** RDS weights for major variables for first 5 weeks (5w) and for all subjects (full) in each survey.

	RDS1 (Full)	RDS1 (5w)	RDS2 (Full)	RDS2 (5w)	RDS3 (Full)	RDS3 (5w)	RDS4 (Full)	RDS4 (5w)
Age								
≤ Median	0.95	0.96	0.92	0.92	0.92	0.92	0.98	0.97
> Median	1.06	1.05	1.09	1.09	1.09	1.09	1.02	1.03
Gender								
Female	1.03	0.80	0.79	0.92	1.08	1.02	0.86	1.00
Male	1.00	1.01	1.01	1.00	1.00	1.00	1.01	1.00
Methadone								
No	1.08	1.07	1.02	1.02	1.04	1.02	1.08	1.01
Yes	0.87	0.87	0.96	0.96	0.92	0.95	0.85	0.98
Methamphetamine								
No	1.08	1.15	0.91	0.91	0.94	0.97	1.09	1.00
Yes	0.96	0.93	1.06	1.06	1.04	1.02	0.87	1.00
HIV								
Negative	1.02	1.00	1.03	1.02	1.05	1.03	1.01	1.01
Positive	0.95	1.00	0.93	0.94	0.87	0.92	0.97	0.98
HCV								
Negative	1.02	0.95	1.02	1.01	0.98	1.03	0.94	0.98
Positive	0.99	1.02	0.99	1.00	1.01	0.99	1.02	1.01

## Discussion

### Value of large scale PWID surveys

There were multiple reasons for a large sample size in the DRIVE study. Not only did we wish to find a low observed HIV incidence, but we needed to show a low upper 95% confidence limit for the observed incidence rate to have statistical confidence in the conclusion of “ending an HIV epidemic.” A large sample size would also allow examination of factors associated with seroconversion for targeting further prevention efforts. (Though with only 3 seroconversions in DRIVE, we were not able to identify factors associated with seroconversion).

Also of considerable importance was the value of a large sample size for convincing policy makers that it is possible to end an HIV epidemic in a key population. The study was conducted during a time of decreasing support for HIV prevention among PWID. Policy makers may not fully grasp concepts such as the upper confidence level for an incidence rate, but are likely to grasp the value of a large sample size within a key population.

The large sample in the surveys in the DRIVE study did provide compelling evidence for very low HIV incidence among PWID in Hai Phong from 2016–2019. HIV recency testing of respondents at intake to the 4 surveys showed 0 recent HIV infections in 1285 PYAR. HIV testing of PWID who participated in multiple surveys showed 0 seroconversions in 696 PYAR. The cohort study which recruited from the surveys found 3 seroconversions in 1483 PYAR. The overall incidence was 3 in 3464 PYAR for and incidence rate of 0.085/100 PYAR, 95% CI 0.02/100–0.25/100.

As a result of the DRIVE findings the Vietnam Administration for HIV/AIDS Control (VAAC) has now started using simplified DRIVE methods for key populations in other provinces. A single large RDS survey is conducted, with HIV recency testing and viral load testing for all HIV seropositives. Incidence from the recency testing and PWID population viral load (percentage of HIV seropositive PWID not at viral suppression) are calculated, and if the criteria for “ending an HIV epidemic” (incidence < 0.5/100 person-years, percentage of the PWID population who are HIV seropositive and not on ART < 5%) are met, prevention resources are focused on maintaining PWID in appropriate services (obtaining sterile syringes, receiving methadone maintenance treatment, receiving and adhering to ART). If the criteria for ending an HIV epidemic have not been met, resources are focused on identifying PWID in need of services and assisting them to utilize services.

### Robustness of RDS to modest changes

We modified the RDS methods by adopting snowball sampling methods—permitting individual participants to recruit large numbers of new participants, up to 20—rather than limiting recruitment to modest numbers (typically 3). We consider the increase of coupons offered for recruiting new participants from 3 to 20 to be an important but still modest modification to RDS methods.

The comparisons of the characteristics of respondents recruited through standard RDS methods versus respondents recruited through snowball methods showed few differences. The comparisons of the RDS analytics (waves to reach equilibrium and homophilies and RDS weights) for the first 5 weeks versus later weeks of recruiting showed minimal differences.

With four replications of these comparisons, we would conclude that the RDS methods were quite robust with respect to our modifications of the standard RDS methods.

### “Representativeness” of the surveys

In our assessment, when the assumptions for RDS are met and the standard RDS procedures are followed, this method is the most likely to generate an unbiased sample of a PWID population. In our surveys, the standard procedure would have been using a limited number of coupons distributed to each respondent. However, we do not believe that our data for either the 5 week or full samples show any epidemiologically meaningful indications of bias in our surveys. As noted above, equilibria for major variables were reached in a small number of waves in each survey. Failure to reach equilibria would be indicative of possible biases in the samples [25]. Similarly, homophilies were low for major variables in each of the surveys. If we had found many statistically significant differences between respondents recruited through regular RDS methods and respondents recruited through snowball methods, this would also suggest possible biases in the samples. The suggestion of bias would be particularly concerning if there had been consistent differences in the same variables across multiple surveys. That we had four replications of our methods also increases confidence in our findings.

And as noted above, the large size of our total sample is an argument for generalizing to the population as a whole. We previously estimated the population of persons actively injecting drugs in Hai Phong as 5000 [7]. With an estimated turnover of 5% per year in the PWID population, this would give 7000 individual PWID during the 4 years of the surveys. We enrolled 4279 individual PWID in the 4 surveys, or 61% of the estimated PWID population during the period of the surveys. PWID who did not participate in the study would have to had a much higher rate of HIV incidence (approximately 6 times higher than the rate among PWID who did participate in the study) for the total incidence to be greater than 0.5/100 person-years in the population as a whole [3].

### Importance of CBOs

Finally, it is important to again note the importance of the CBO staff in conducting the surveys. They participated in research decisions and data collection and provided support and assistance to the participants in the study. Their ability to develop trusting relationships with the thousands of respondents was crucial to the smooth function of the surveys, and their expertise was critical in the decision to incorporate snowball sampling into the RDS recruitment.

### Limitations

The major limitation of the study was, of course, our inability to utilize standard RDS procedures throughout the entirety of each of the surveys. As noted in the Methods, from our knowledge of the drug use situation in Hai Phong, we believe that the main factor reducing RDS recruitment was the increase police surveillance of the drug distribution “hotspots.” We did directly observe several hotspots. Many persons who appeared to be PWID would come to a hotspot, quickly transact some business and then leave. They did not stay the 10 minutes or so that would be needed to discuss the study and recruit or be recruited to participate in the study.

Our adaptation to this problem was to increase the numbers of coupons given to participants who stated that they would be able to recruit sizable numbers of new participants, in effect, adding snowball sampling to our recruitment methods. We selected this modification because our discussions with respondents indicated that this would be the most likely means of reaching our desired sample sizes. An alternative modification would have been to select new seeds and initiate new RDS recruitment chains. This is the modification used in the CDC National HIV Behavior Surveys when recruitment slows across all chains [26]. It would have been an interesting methodological experiment to have alternated between the snowball sampling and the recruiting new seeds methods of adapting to the slowed recruiting for direct comparison. Once we learned that the snowball adaptation permitted us to get very close to our desired sample size, however, the importance of maintaining consistent methods in the overall study precluded such a methodological experiment. As noted in Methods, we relied upon the core RDS assumption that recruiters randomly recruited from within their injecting networks to classify standard RDS recruits from snowball recruits for persons who recruited more than 3 new respondents. If this assumption was not valid, then the RDS analytics would not be valid for either the standard RDS participants or the snowball participants.

A second limitation was that we did not include syringe sharing as one of the major variables in our applications of the RDS analytics despite its obvious relevance to HIV transmission among our respondents. As noted in the Methods, the major variables selected for use with the RDS analytics were variables for which social desirability bias would not have had a major effect. We suspect that social desirability bias was a factor in the extremely low percentages of self-reported syringe sharing and did not believe that we would be able to separate the social desirability bias from the reported syringe sharing reports.

A third limitation concerns PWID who decided not to participate in the surveys. Our respondents reported that there were multiple reasons given for not wanting to participate, including concerns about confidentiality for their drug use and/or HIV status and other demands on their time, such as employment. As noted in the above comments concerning the large sample size, however, the HIV incidence would have to be substantially higher among these not-willing-to-participate PWID (approximately 6 times higher) to invalidate our conclusion that the HIV incidence was 0.5/100 PYAR or less.

Despite these limitations, we do believe that our methodological analyses do demonstrate that modified RDS recruiting can be used to obtain large sample sizes for PWID surveys, and that RDS is robust with respect to the modification of adding a moderate amount of snowball recruiting.

## Conclusions

Large sample sizes may be needed to support conclusions about “ending an HIV epidemic” in a key but hard to reach population such as PWID. We used respondent driven sampling supplemented by snowball sampling to recruit large sample sizes—approximately 30% of the local PWID population in each of four annual surveys. Comparisons of participants recruited by RDS and participants recruited by snowball sampling showed minimal differences and examination of RDS analytics produced no indications that the snowball sampling introduced bias. RDS appears to be relatively robust with respect to modest modifications such as adding snowball sampling for 20% of total subjects. The four replications of the analyses lend considerable support to these conclusions.
